# New and Emerging Systemic Therapeutic Options for Advanced Cholangiocarcinoma

**DOI:** 10.3390/cells9030688

**Published:** 2020-03-11

**Authors:** Sara Massironi, Lorenzo Pilla, Alessandra Elvevi, Raffaella Longarini, Roberta Elisa Rossi, Paolo Bidoli, Pietro Invernizzi

**Affiliations:** 1 Division of Gastroenterology, San Gerardo Hospital, University of Milano-Bicocca School of Medicine, 20900 Monza, Italy; alessandra.elvevi@gmail.com (A.E.); pietro.invernizzi@unimib.it (P.I.); 2 Division of Medical Oncology, San Gerardo Hospital, University of Milano-Bicocca School of Medicine, 20900 Monza, Italy; lopilla@me.com (L.P.); r.longarini@hsgerardo.org (R.L.); p.bidoli@asst-monza.it (P.B.); 3 Gastrointestinal and Hepato-Pancreatic Surgery and Liver Transplantation Unit, Fondazione IRCCS Istituto Nazionale Tumori (INT, National Cancer Institute) - Università degli Studi di Milano, 20100 Milan, Italy; robertaelisa.rossi@gmail.com

**Keywords:** cholaniocarcinoma, molecular landscape, targeted-therapy, immunotherapy, chemotherapy

## Abstract

Cholangiocarcinoma (CCA) represents a disease entity that comprises a heterogeneous group of biliary malignant neoplasms, with variable clinical presentation and severity. It may be classified according to its anatomical location and distinguished in intrahepatic (iCCA), perihilar (pCCA), or distal (dCCA), each subtype implying distinct epidemiology, biology, prognosis, and strategy for clinical management. Its incidence has increased globally over the past few decades, and its mortality rate remains high due to both its biological aggressiveness and resistance to medical therapy. Surgery is the only potentially curative treatment and is the standard approach for resectable CCA; however, more than half of the patients have locally advanced or metastatic disease at presentation. For patients with unresectable CCA, the available systemic therapies are of limited effectiveness. However, the advances of the comprehension of the complex molecular landscape of CCA and its tumor microenvironment could provide new keys to better understand the pathogenesis, the mechanisms of resistance and ultimately to identify promising new therapeutic targets. Recently, clinical trials targeting isocitrate dehydrogenase (IDH)-1 mutations and fibroblast growth factor receptor (FGFR)-2 fusions, as well as immunotherapy showed promising results. All these new and emerging therapeutic options are herein discussed.

## 1. Introduction

Cholangiocarcinoma (CCA) is a group of epithelial cell malignancies arising from cholangiocellular epithelium, most of which are adenocarcinoma [1]. It is a rare cancer, accounting for less than 1% of all human cancers, and around 10%–15% of all primary liver cancers, and it is mostly diagnosed in the seventh decade with a small male predominance (male: female ratio of 1.2—1.5:1.0) [2].

CCA is subclassified as intrahepatic cholangiocarcinoma (iCCA), originating from the biliary tree within the liver, and extrahepatic cholangiocarcinoma (eCCA), outside the liver parenchyma; the latter is further subdivided into perihilar cholangiocarcinoma (pCCA or Klatskin tumor) and distal cholangiocarcinoma (dCCA), with a frequency of 10–20% iCCA, 50% pCCA and 30–40% dCCA [2].

Regarding CCA risk factors, data from literature showed both well-known risk factors and newly emerging ones. In detail, pre-existing medical conditions such as choledochal cysts (i.e., Caroli’s disease), inflammatory bowel disease and primary sclerosing cholangitis are very well-known risk factors. Liver flukes (i.e., clonorchis sinensis and opisthorcis viverrini) represent risk factors with relevant epidemiological influence in some countries (i.e., Korea, Thailand) [3].

The increasing incidence of CCA in the past few decades in Western Countries [2] has suggested looking for emerging risk factors. Among these, cirrhosis, hepatitis C infection, hepatitis B infection, metabolic syndrome and diabetes resulted as risk factors for CCA development, while data about obesity, alcohol and tobacco use are still controversial [3,4].

The best diagnostic tool for CCA is magnetic resonance imaging (MRI) with MR cholangiopancreatography (MRCP), contrast-enhanced and diffusion-weighted imaging, while computed tomography (CT) is generally less useful. Pathology diagnosis should be obtained before any non-surgical treatment modality, while is not essential in patients planned for curative surgery where radiological features are characteristic. When needed, endoscopic retrograde cholangiopancreatography (ERCP)-guided biopsies are preferred to biliary brush cytology and should be carried out whenever possible. Endoscopic ultrasound (EUS)-guided fine-needle aspiration (FNA) is also useful for obtaining micro-specimens, especially when ERCP-guided brush cytology or biopsies are negative or inconclusive [2]. Updated World Health Organization (WHO) classification [5] underlines the relevance of immunohistochemistry to identify different CCA pathological subtypes, which may potentially request a different therapeutic approach. 

Cancer antigen 19–9 (CA 19–9) is the primary serum biomarker used in the diagnosis of CCA and CA 19–9 levels >1000 U/mL have been associated with the presence of metastatic disease involving the peritoneum [1]. In patients with primary sclerosing cholangitis the most reliable cutoff for iCCA is 129 U/mL, even if more than 30% of patients with primary sclerosing cholangitis with a CA 19–9 value higher than 129 U/mL do not have CCA, being as this increases often due to bacterial cholangitis [1].

Once the diagnosis has been obtained, staging is needed. Staging has to take into account the patient’s performance status according to European Cooperative Oncology Group (ECOG) scores, past medical history/co-morbidities and liver function tests (LFTs). Imaging consists of MRI (for assessment of tumor-stage, bile duct, and vascular involvement), thorax CT scan (metastases-stage) and EUS (lymph node-stage). Positron emission tomography (PET)-CT has limited diagnostic value and it should be used on a case-by-case basis [2].

The best therapeutic approach for iCCA is surgical resection with lymphadenectomy at the level of the hepato-duodenal ligament, when feasible [1,2]. Curative surgical resection with negative tumor margins can be achieved in less than 30% of patients [6]. The median survival time by intention-to-treat analysis of lesions considered to be surgically resectable on imaging studies is 36 months [7]. Adjuvant therapy (radiotherapy, chemoradiotherapy or chemotherapy alone) may be offered to patients only after risk-benefit assessment, even if the evidence is weak [2].

Around 60%–70% of patients are diagnosed with advanced stage, i.e. unresectable or metastatic disease [8]; in these cases, systemic chemotherapy is usually the only treatment option; however, since iCCA preferentially spreads to the liver, the loco-regional approach, including trans-arterial chemo-embolization (TACE) and trans-arterial radio-embolization (TARE) might represent a viable option, although solid data are still lacking [1,8]. In this setting (unresectable advanced disease) new emerging therapies are under evaluation. The advances in iCCA biology comprehension, i.e., complex molecular landscape, and tumor microenvironment are the basis for new drugs acting on new therapeutic biological targets. Recently, clinical trials targeting isocitrate dehydrogenase (IDH)-1 mutations and fibroblast growth factor receptor (FGFR)-2 fusions, as well as immunotherapy, showed promising results. Molecular profiling of tumors represents the mainstay for precision oncology. This review provides an overview of the current literature according to the new therapeutic approaches to unresectable/advanced iCCA, with their pearls and pitfalls, giving special attention to the new molecules which have opened a new horizon in the field of treatment of CCA.

## 2. Chemotherapy

### 2.1. Adjuvant Chemotherapy

Treatments after radical surgery for biliary tract cancer include chemotherapy, radiotherapy, and the combination of the two. The role of post-operative treatment is somewhat unclear due to conflicting results of randomized trials. The first study demonstrating a benefit of chemotherapy with mitomycin and 5-Fluorouracil in the gallbladder group in terms of overall survival and progression-free survival compared to observation, was published in 2002 [9]. ESPAC-3 trial was another positive study that evaluated the efficacy of 5-Fluoruracile or gemcitabine compared to observation in periampullary carcinoma, with a better toxicity profile for gemcitabine [10]. The meta-analysis by Horgan et al. evaluated data from more than 6000 patients who underwent different types of post-surgical treatments (chemotherapy, radiotherapy, radio-chemotherapy): despite the intrinsic limitations of this study, the analysis confirmed the benefit of adjuvant chemotherapy and chemoradiotherapy, especially in patients with node-positive and surgical positive margin [11]. Moreover, this analysis showed the lack of benefit of radiotherapy in patients with negative surgical margins (R0 resection).

Another recent meta-analysis of 30 studies confirmed these results, concluding that adjuvant chemotherapy implies a 41% reduction in the risk of death, but the benefit is inferior when post-operative radiotherapy is associated with chemotherapy [12].

Following this meta-analysis, the results of other three randomized phase III studies became available. In the PRODIGE12-ACCORD18-study, 193 patients were randomized to observation or GEMOX scheme (Gemcitabine/oxaliplatin) after surgery: no significant differences were seen between the two arms in terms of relapse-free survival [13]. Again, also the BCAT study failed to show benefit for gemcitabine therapy [14].

On the other hand, the BILCAP-study compared 8 cycles of capecitabine to surveillance: in 447 patients, the median overall survival was 36.4 months for the control group, and 51.1 months in the experimental arm (HR 0.81 95% CI 0.63–1.04 *p* = 0.097), reaching the statistical significance after the correction for prognostic factors [15]. Based on these data, capecitabine has evolved as the new standard of care after curative resection of biliary tract cancer and capecitabine became the control arm in ongoing emended phase-III trial, the ACTICCA-1 study, in which the experimental arm is represented by cisplatin/gemcitabine (NCT02170090) [16].

### 2.2. Chemotherapy for Metastatic Disease: First and Second Lines

Since the publication of the pooled analysis by Eckel et al. [17], we know that the combination of chemotherapy, in particular, the association of platinum-compounds with gemcitabine, is superior to monotherapy in the metastatic setting.

Based on the convincing data of the AC-02 trial, the current standard first-line treatment for CCA not suitable for surgery or loco-regional treatment is the combination of gemcitabine and cisplatin [18]. The trial demonstrated a higher median overall survival for the combination arm compared to gemcitabine monotherapy (11.7 vs. 8.1 months, respectively; hazard ratio 0.64; 95% CI 0.52–0.8; *p* < 0.001). Moreover, the disease control rate was 81.4% for the combo and 71.8% for monotherapy.

Similar results are reported in the Japanese phase II BT22 trial [19] and confirmed by the meta-analysis by Valle and colleagues [20].

Trials investigating the combination of gemcitabine with oxaliplatin demonstrated a median overall survival ranging from 8.3 to 12.4 months with overall response rate which varies from 15% to 50% [21,22], with a more favorable toxicity profile for oxaliplatin than cisplatin.

Additionally, fluoropyrimidine-based chemotherapy has shown efficacy in advanced biliary tract cancers [23,24], but a direct comparison between gemcitabine-based and fluoropyrimidine-based regimens is lacking.

The most important independent prognostic factor for advanced biliary tract cancer is the performance status (PS) ECOG [25], which can guide therapeutical choices. Indeed, in patients with PS ECOG 2 monotherapy should be preferred.

Another unanswered question is whether more intensive treatment is superior to a two-drug’ s standard combo. Some interesting trials addressed this issue, such as the aBTCs trial, a phase II trial focused on triplet therapy cisplatin, gemcitabine and nab-paclitaxel [26], as well as the phase III trial of cisplatin, gemcitabine plus S1 [27].

An interesting approach, in this context, is represented by the application of ProTide technology to gemcitabine. Acelarin (NUC-1031), a phosphoramidate transformation of gemcitabine, is a first-in-class nucleotide analogue which showed, in pre-clinical models, to modify the transport, activation, and catabolism of gemcitabine, thus allowing to overcome some crucial resistance mechanisms [28]. Currently, a phase III trial, which compares acelarin plus cisplatin to gemcitabine plus cisplatin as a first-line treatment of biliary cancer is ongoing (NuTide trial) [29].

When patients show cancer progression after first-line chemotherapy, a good PS ECOG is the most important selection factor for the activation of second-line therapy [30].

A systematic review of several trials (phase II trials, retrospective trials) by Lamarca et al. explored the clinical benefit of treating with second-line therapy patients who progressed after first-line chemotherapy. The treatment schedules used were fluoropyrimidine, irinotecan, docetaxel, gemcitabine and platinum-compounds if fluoropyrimidines were used as first-line chemotherapy. The review demonstrated a calculated median overall survival of about 6.6 months when analyzing phase II trials and 7.7 months when retrospective trials were considered. Moreover, median progression-free survival was 2.8 months and the median response rate was only 7.7%, without clear evidence of benefit in recommending second-line chemotherapy in all patients [31].

The first randomized phase III study ABC-06 randomized 162 patients to active symptom control (i.e., antibiotic therapy, corticorticosteroid therapy, biliary drainage) and FOLFOX regimen (oxaliplatin/fluorouracil) after cisplatin-gemcitabine failure. Although the reported median survival benefit of FOLFOX regimen over active symptom control was small (5.3 versus 6.2 months, adjusted HR 0.69), the FOLFOX regimen obtained more significant survival rate at 6 (35.5% versus 50.6%) and 12 months (11.4% versus 25.9%) [32]. The available studies globally support the use of second-line therapy in young and fit patients. There are some ongoing trials focusing on second-line treatment. An active drug used as a second-line treatment is nal-IRI (liposomal irinotecan), which is compared to fluorouracil in a phase II ongoing german trial (NCT03043547). Some phase-III studies are still ongoing, mainly exploring the role of gemcitabine and capecitabine as a second-line CCA treatment. Finally, the phase III trial TreeTopp compares capecitabine with or without Varlitinib, a tyrosine kinase inhibitor targeting epidermal growth factor receptor (EGFR) and human epidermal growth factor 2 e 4 (HER2 and HER4) (NTC03093870). A summary of available phase-III studies regarding chemotherapy for CCA is reported in Table 1.

## 3. Targeted Therapies

**Figure 1:**
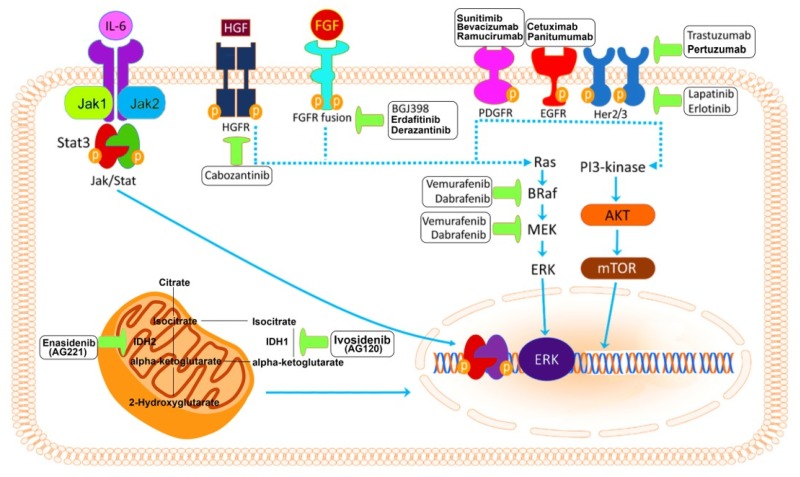
The complex molecular horizon of biological targets in cholangiocarcinoma. Adapted from Simile et al.

Several recent studies identify key oncogenic drivers as possible targets and compounds are tailored to the targets (Figure 1).

**Figure 2:**
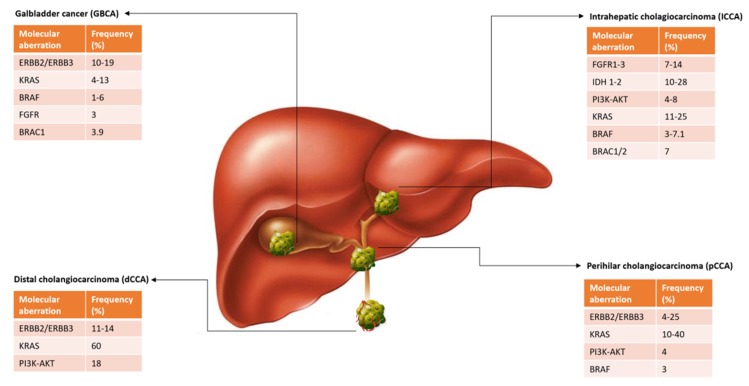
Distinct patterns of molecular mutations recurring in specific anatomic subtypes of cholangiocarcinoma.

The marked inter-tumoral and intra-tumoral heterogeneity of CCA has contributed to the lack of effective targeted therapies for this disease. Moreover, in most clinical trials, investigators have grouped together patients with different subtypes of the disease, under the broad definition of ‘biliary tract cancer’, rather than stratifying patients according to the presence of relevant oncogenic drivers. Molecular profiling studies have better delineated the genetic landscape of each CCA subtype, highlighting distinct patterns of mutations recurring in specific anatomic subtypes [33,34] (Figure 2). Alterations of isocitrate dehydrogenase (IDH)1, IDH2, fibroblast growth factor receptor (FGFR)1, FGFR2, FGFR3, epoxide hydrolase (EPH)A2, and biofilm-associated surface protein (BAP)1 genes have been reported in the intra-hepatic subtype, while in perihilar and dCCA genetic alterations of AT-rich interactive domain (ARID)1B, E74-like factor (ELF)3, protein polybromo-1 (PBRM1), protein kinase cAMP-activated catalytic subunit alpha (PRKACA), and PRKACB were described [34]. The distinguished genetic profile, histological features and clinical outcomes reported in these different anatomical sites could lead one day to tailored treatment approaches.

### 3.1. Targeting Mutations of Isocitrate Dehydrogenase (IDH) 1 and 2

Mutations of IDH1 and 2 frequently occur in iCCA [35]. IDH catalyzes the conversion of isocitrate to α-ketoglutarate. Alterations of IDH, through the accumulations of oncometabolites, induces widespread epigenetic changes that have a pleiotropic effect on differentiation, cell growth, and hypoxia signaling [36]. Approximately 14% of iCCA tumors are known to harbor IDH genetic mutations. IDH mutations are less frequently observed in pCCAs and dCCAs [37]. IDH1 mutations are more common than IDH2. Different inhibitors specific to IDH-mutant alleles have been developed. Inhibitors of IDH1 (AG120, IDH305), IDH2 (AG221), and pan-IDH1/2 (AG881) are currently being tested in patients with iCCA.

#### 3.1.1. Ivosidenib

AG-120 (Ivosidenib) was tested in 73 patients with IDH1-mutant advanced CCA in a phase I study. Four (5%) patients had a partial response, 56% experienced stable disease, and the median overall survival was 13·8 months. Results of the cross-over phase III study (ClarIDHy) of Ivosidenib compared to placebo were reported at ESMO 2019. Ivosidenib significantly improved PFS compared with placebo. The median OS was 10.8 months for Ivosidenib and 9.7 months for placebo, with 57% of placebo patients crossing over to Ivosidenib. In the intention to treat population, there was a trend in favor of Ivosidenib, but it was not yet significant [38,39].

Despite the cross-over design hampers the possibility to show significant overall survival data, this remains a landmark study providing level A evidence for the efficacy of targeted therapy in CCA and establishes the role of molecular profiling in this cancer. 

#### 3.1.2. Enasidenib

AG-221 (Enasidenib), a selective inhibitor of mutant IDH2, has demonstrated activity in pre-clinical models of acute myeloid leukemia (AML) [40,41,42] and is currently being assessed in multiple phases I/II clinical trials in subjects with advanced solid tumors, including iCCA, who harbor an IDH2 mutation (NCT02273739).

Other IDH1 and IDH2 inhibitors are also now in clinical trials (NCT02273739, NCT02381886, and NCT02481154), mainly including patients with iCCA.

### 3.2. Targeting FGFR

The discovery of FGFR alterations in multiple tumor types has boosted scientific interest in the development of FGFR inhibitors. In iCCA recurrent FGFR2 fusions are found in 11% to 45% of patients [43,44]. FGFR2 fusions result in constitutive tyrosine kinase activity [45], which in turn led to downstream signaling pathways activation, such as RAS-RAF-MEK.

However, the mechanisms by which FGFR displays its oncogenic activity are not fully described yet.

#### 3.2.1. Infigratinib

BGJ398 (Infigratinib; Novartis AG) is an oral non-selective FGFR inhibitor, assessed in a phase II trial in patients with different FGFR alterations [FGFR2 fusions (*n* = 48), FGFR2 mutations (*n* = 8), FGFR2 amplification (*n* = 3)] after first-line chemotherapy. The overall response rate was 14.8%, almost all with FGFR2 fusions, and median progression-free survival was 5.8 months, and interestingly disease control rate was 75.4%; however, the durability of response was limited [46]. Currently, a phase III clinical trial is evaluating BGJ398 versus chemotherapy with Cisplatin and Gemcitabine in first-line treatment in patients with locally advanced/metastatic CCA with FGFR-2 gene fusions/translocations (NCT03773302).

Goyal and colleagues performed an integrative analysis in three patients treated with BGJ398 who developed acquired resistance. Analysis of cell-free circulating tumor DNA (cfDNA), primary tumors, and metastases showed the emergence of multiple recurrent point mutations of FGFR2 at disease progression. Of note, other structurally different FGFR inhibitors demonstrated to overcome specific FGFR resistance mutations in vitro [47].

#### 3.2.2. Erdafitinib

Erdafitinib (JNJ-42756493, Jansenn^®^) is a second pan-FGFR small molecule kinase inhibitor being tested in clinical trials. In a phase I study Erdafitinib showed anti-tumor activity only in the 21 patients with FGFR mutations, while 36 patients that did not have confirmed FGFR mutations had no significant response [48]. These results were further confirmed by a recent publication, in which Erdafitinib was evaluated in a phase Ib basket trial. In the CCA cohort, 3 out of 11 patients with FGFR mutations or fusions had a partial response [49].

#### 3.2.3. Derazantinib

Derazantinib (DZB)—an orally bioavailable, multikinase inhibitor with potent pan FGFR activity—is currently under evaluation in several clinical phase 2 trials for iCCA. DZB inhibited the growth of CCA cell lines in a dose-dependent manner, and extracellular signal-regulated kinase 1/2 and AKT. It also activated apoptotic and cell growth arrest signaling. DZB reduced the in vitro invasiveness and the expression of key epithelial-mesenchymal transition genes [50]. The in vitro data correlated with the expression of FGFRs in human CCA specimens by immunohistochemistry and the CCA cell lines assayed by Western blot analysis.

Interestingly, in pre-clinical models, DZB demonstrated to be active in tumors with FGFR alterations, including fusions, amplifications, and mutations [50]. A multicenter, phase I-II clinical trial open-label study enrolled adult patients with unresectable iCCA with FGFR2 fusion, who progressed, were intolerant or not eligible to first-line chemotherapy. Overall response rate was 20.7%, and disease control rate was 82.8% [51]. As for the other FGFR inhibitors, the treatment was well tolerated with a manageable safety profile. Hyperphosphatemia is a specific on-target side effect of this class of compounds, owing to increased renal phosphate re-absorption caused by the inhibition of FGF23 [52]. Other side effects include decreased appetite, diarrhea, constipation, ocular toxicity, and mucosal dryness.

Additional FGFR-selective inhibitors, such as TAS-120 (NCT02052778), Debio 1347 (NCT01948297), Pemigatinib (NCT02924376, NCT02393248) and Ponatinib, (NCT02265341) are currently in early phase clinical trials in patients with advanced-stage solid-organ malignancies, including iCCA.

### 3.3. MAPK (Mitogen-Activated Protein Kinases) Pathway

Mutations of BRAF are rare but occur mostly in iCCA, with a prevalence of 1%–3% [53]. BRAF mutations at codon 600, mostly V600E, are of interest because they are potentially targetable with BRAF inhibitors. In a phase II basket trial with Vemurafenib, only one patient out of 12 with iCCA demonstrated a partial response [54]. The limited activity of single-agent BRAF inhibitors might be due to feedback EGFR activation as in colorectal cancer. The inhibition of MEK could be an alternative strategy to target MAPK. A study with selumetinib in advanced CCA showed evidence of anti-tumor activity, with three partial responses out of 25 patients (12%) and 17 disease stabilizations. The median progression-free survival was 3.7 months (95% CI, 2.1–11.2), and the median overall survival was 9.7 months [39].

The dual inhibition of BRAF and MEK is an alternative and potentially more efficient strategy to target the RAS-ERK pathway. In two independent reports, the combination of Dabrafenib and Trametinib showed durable clinical responses [55,56]. Finally, the preliminary results of a basket trial involving patients with BRAF mutation showed, in a cohort of pretreated biliary tract cancer, a response rate of 42% with a median overall survival of 11.7 months [57].

### 3.4. Agents Targeting HER FAMILY (ERBB2) Receptors

Two major classes of anti-ERBB therapies are used in cancer, which are monoclonal antibodies, blocking ligand binding, and tyrosine kinase inhibitors (TKIs), which target the catalytic domain of the receptor.

Alterations of epidermal growth factor receptor (ERBB) family have been reported in CCA, mostly in gallbladder cancer (19%) and in pCCA/dCCA (17%) [43], as compared to iCCA (4.8%) [58]. While the pathophysiological role of ERBB3 and ERBB4 in CCA is still unknown, several studies have described the tumorigenic role of EGFR and ERBB2 in CCA, which is mediated by the activation of MAPK-ERK or PI3k-mTOR pathways [59]. Different EGFR inhibitors have been tested in CCA either as a single agent or in combination, mostly in KRAS wild type tumors. Erlotinib alone, or in combination with cetuximab, demonstrated limited clinical activity. Panitumumab, combined with gemcitabine and irinotecan, showed promising results. However, in a phase II trial, panitumumab combined with oxaliplatin and gemcitabine did not display any advantages over gemcitabine and cisplatin alone. Overall, these data failed to support further development of EGFR inhibitors in this setting. Similarly, current data on HER2 directed therapy in gallbladder cancer (GBC) are contradictory and evidence of efficacy are limited to retrospective case reports or case series [59,60,61], while more recent earlier investigations with HER-2 directed therapy in unselected populations failed to show activity in advanced CCA [62,63]. Conversely, one study performed in a cohort of 8 patients with either overexpression or gene amplification resulted in an interesting clinical activity, with 1 complete response, 3 partial responses and 4 disease stabilities [59]. These results are in line with other case reports describing the remarkable activity of trastuzumab treatment in HER2-positive gallbladder cancer patients [60,64,65]. Future prospective studies in selected populations will help to define the role of mAB and small molecule TKIs directed to HER-2, as a single agent or in combination with chemotherapy, in the treatment of CCA.

### 3.5. ROS1 and Neurotrophic Tyrosine Kinase Receptor (TRKA)

ROS1 kinase fusion proteins have been reported in a subset of CCA (8.7%) [66]. The pre-clinical model supports the oncogenic role of FIG–ROS1 fusion in iCAA [67], and its potential therapeutic target in CCA. The ALK and ROS1 inhibitor ceritinib and crizotinib are currently being evaluated in two phases II in patients with advanced CCA (NCT02374489, NCT02034981)

NTRK gene fusions can drive unregulated cell growth and proliferation in a range of cancer types. Recently, this pathway gained significant focus and attention in precision oncology. Larotrectinib and entrectinib are first-generation TRK inhibitors and have demonstrated rapid and durable responses and favorable safety profiles in patients with TRK fusion-positive cancers. Even if only few cases of CCA are included in a current basket trial evaluating Entrectinib, preliminary results are encouraging [68]. Therefore, Entrectinib is now under evaluation clinical trials in patients harboring ROS1 ALK fusions (NCT02568267) or TRKA (NCT02568267).

### 3.6. Targeting BRCA and BRCA Associated Protein

It is known by recent findings that the presence of germline mutation of BRCA1 and BRCA2 confers an increased lifetime risk of developing CCA. The Breast Cancer Linkage Consortium reported an estimated relative risk for in BRCA2 mutation carriers of 4.97. Churi and colleagues [43] reported in a significant proportion of CCA alterations affecting genes involved in DNA repair pathways.

Cancers harboring these types of mutations are sensitive to DNA damaging therapies [69] and to poly ADP ribose polymerase (PARP) inhibition [70]. Golan and colleagues treated four patients with BRCA mutated CCA with PARP inhibitors and obtained a favorable progression-free survival and overall survival [71].

Few studies of PARP Niraparib and olaparib are currently ongoing in patients with CCA, and with aberrant DNA genes mutations (NCT04042831, NCT03207347).

### 3.7. Angiogenesis and Non-Selective Kinase Inhibitors

Several anti-angiogenic inhibitors have been tested in clinical trials. This approach is supported by the evidence that multiple angiogenic factors and their respective receptors are present either in biliary tract cancer or in its microenvironment [72,73]. Moreover, factors associated with angiogenesis have prognostic significance [74,75]. In two different phase II trials, bevacizumab was evaluated in combination with gemcitabine and oxaliplatin in advanced CCA, showing signs of activity and prolongation of progression-free survival as compared to gemcitabine and oxaliplatin alone, albeit the differences were not significant [76,77]. Similar results were obtained with an oral vascular endothelial growth factor receptor (VEGFR)1-VEGFR2-VEGFR3 TKI cediranib [78].

Sorafenib, a multi-kinase and angiogenesis inhibitor, showed an interesting inhibitory activity in a pre-clinical model of CCA, although these premises did not translate into clinical activity. Similar results were observed with sunitinib and regorafenib [79].

### 3.8. Other Target Agents in Early Clinical Development

Promising new agents in early clinical development for the treatment of CCA include compounds that target the JAK/STAT pathway, the Wnt/β-catenin signaling and the Hedgehog signaling (HH) pathways. JAK/STAT pathway activation is directly involved in several cellular process characteristics of cancer cells, including cell growth, proliferation, and apoptosis [80,81,82].

Dysregulated JAK/STAT activation has been detected in 50% of patients with CCA, especially with an inflammatory microenvironment. STAT-3 activation is increased more frequently in iCCA. Several inhibitors of JAK/STAT are already approved for the treatment of myelofibrosis and are currently under investigation in different cancers.

In a phase I trial the sphingosine kinase inhibitor, ABC294640 (Yeliva^®^), which also inhibits STAT3 phosphorylation, showed activity also in cholangiocarcinoma (NCT01488513). Phase II studies in different tumor histologies, including cholangiocarcinoma, are actively recruiting patients (NCT03377179, NCT03414489).

The involvement of Wnt/B-catenin signaling in cancer cell regulation, invasion, and migration, makes it a promising pathway for drug targeting the β-catenin expression has been detected in CCA [83]. In a pre-clinical model of CCA, blocking of WNT resulted in increased apoptosis, cell cycle arrest and chemoresistance [84]. Multiple WNT pathway inhibitors are currently under clinical development (see Table 2). The activation of the hedgehog pathway results in chronic hepatic inflammation, fibrosis, cholangiopathies and in the development of CCA [80,83]. In cancer specimens of CCA, the expression of Hedgehog pathway components have been associated with disease stage and prognosis [85]. In pre-clinical models of CCA different HH inhibitors showed anti-tumor activity, especially in association with chemotherapy, by increasing intratumoral vascularization and drug delivery [86].

MET overexpression and amplification have been described both in intrahepatic (12%–58%) and perihilar/distal cholangiocarcinoma (16%) [87] and is associated with shorter survival [84]. However, clinical results with MET inhibitors monotherapy were discouraging, while the combination with chemotherapy seemed to be more promising [88].

NOTCH pathway is also considered another attractive target for cholangiocarcinoma therapy. Notch signaling is implicated in the differentiation of cholangiocyte lineage [89], increased proliferation and survival of CCA cells, and is associated with a worse prognosis [80].

Finally, an unconventional target in CCA is represented by cancer-associated fibroblasts (CAF). CAFs often outnumber tumor cells, contribute to CCA development by the production of tumor stroma, and the secretion of soluble factors, involved in the neoplastic process [90]. Inhibition of CAFs activity has already shown anti-tumor activity in pre-clinical models [91]. Currently, the development of agents targeting CAFs is ongoing only in non-oncologic indications.

## 4. Immunotherapy for Cholangiocarcinoma

The immune system has the extraordinary capability of detecting and killing aberrant cells but is regulated by a complex network of immune-checkpoint proteins. Modulation of the local immunosuppressive tumor microenvironment has emerged as a possible mechanism to get antitumor activity in a variety of tumor types. Huge progress has been recently made in the understanding of how cancer evades the immune system, which in turn offers new ways to stop cancer immune evasion in favor of eliminating cancer cells [92]. The utilization of these path-ways is an important mechanism of immune evasion of cancer cells. Cancer immunotherapy is based on the utilization of monoclonal antibodies targeting these immune checkpoint regulators which can increase endogenous anti-tumoral activity [93].

### 4.1. Checkpoints Inhibitors (ICIs)

The well-known immune checkpoints inhibitors (ICIs) targeting the programmed cell death 1 (PD-1) or the cytotoxic T-lymphocyte associated antigen 4 (CTLA-4) checkpoints have demonstrated the potential for relatively tumor-specific immune disinhibition. According to data from the literature, inhibition of immune checkpoints has shown promising results in several malignancies such as melanoma [94], non-small cell lung cancer [95], urothelial carcino-ma [96], renal-cell carcinoma [97], head and neck cancer [98] and hepatic cancer [99].

At present, the clinical data on immunotherapy in CCA and other biliary tract cancers are limited and several trials are ongoing exploring, for instance, the role of monoclonal antibodies ipilimumab or tremelimumab (anti- CTL4) or antibodies targeting PD-L1, such as durvalumab, or its receptor programmed cell death protein 1 (PD-1), such as pembrolizumab or nivolumab [33,100].

In small studies on CCA tumor samples, PD-L1 expression has been reported in 9%–72% of specimens [101,102,103], and on 46%–63% of immune cells within the tumor microenvironment [101,102,103]. These data indicate that a substantial proportion of CCAs might be amenable to therapy with PD-1 or PD-L1 inhibitors. Moreover, Tumor MMR protein deficiency, which results in the genetic signature of microsatellite instability (MSI), with high rates of somatic mutation and increased expression of tumor-associated antigens, predicts responsiveness to ICI across tumor types. In a whole-exome-sequencing study of 231 CCA tumor samples [34], a median of 39 and 35 somatic non-synonymous mutations were identified in intrahepatic and extrahepatic CCAs, respectively; overall, ~6% of the CCAs had evidence of hypermutation, with concurrent MMR deficiency and/or MSI detected in about 36% of this hypermutated tumors. Accordingly, a recent review of published studies reported up to 10% of iCCAs with MSI or MMR deficiency [104], although other studies suggest a lower frequency. Together, these data suggest that immune-checkpoint blockade and immune-modulating therapies could be promising options for the subgroup of patients with CCAs harboring high mutational loads.

#### 4.1.1. Pembrolizumab

The anti-PD-1 antibody pembrolizumab has been approved by the United States Food and Drug Administration for previously treated patients with DNA mismatch repair (MMR) deficiency and/or microsatellite instability (MSI)-high advanced solid tumors, independent of histology, which would include those with CCA. Of note, MMR deficiency has been reported to occur in 5% to 10% of CCAs [104]. Pembrolizumab is a highly selective, humanized monoclonal antibody against PD-1 that is designed to block the interaction between PD-1 and its ligands, PD-L1 and PD-L2.

KEYNOTE-028 (ClinicalTrials.gov, NCT02054806) is another ongoing, multi-cohort, phase 1b trial of pembrolizumab monotherapy for patients with PD-L1-positive advanced solid tumors, including PD-L1-positive adenocarcinoma of the gallbladder or biliary tree, excluding cancer of the ampulla of Vater. Interim safety and efficacy data have been reported for a small cohort of patients with PD-L1-positive biliary tract cancer; 37 of 89 patients screened (41.6%) had PD-L1 expression on ≥1% of tumor cells by immunohistochemistry, 24 of whom enrolled in the study (20 with CCA, four with gallbladder carcinoma) [101] Pembrolizumab 10 mg/kg was given every two weeks for up to two years or until confirmed progression or unacceptable toxicity. Pembrolizumab was generally well tolerated and demonstrated promising antitumor activity as four (17%, three with CCA and one with gallbladder carcinoma) out of the 24 patients had a partial response, and four (17%) had stable disease. The duration of partial response was protracted, with the median PFS not reached at the time of reporting. The rate of grade 3 toxicities was 16.7%, with no patients experiencing grade ≥4 toxicities, nor any marked hepatotoxicity [101].

In view of the promising safety and efficacy of pembrolizumab in the KEYNOTE-028 biliary cancer cohort, another trial including a biliary cancer cohort of 104 patients is ongoing (KEYNOTE-158 basket trial). To date, KEYNOTE-158 (NCT02628067) is the largest study with pembrolizumab, including patients with advanced biliary cancers without known MMR deficiency, after progression on or intolerance to at least one line of standard therapy. Among 104 patients, the measured overall response rate was 5.8%, but the tumor mutation status and the proportions of patients with iCCA vs. eCCA or gallbladder carcinoma were not reported and the rate of CCA MSI status was mainly “non-high” (95.2%) with none of the CCA showing MSI-high [101]. In another partial analysis from the same study, Marabelle et al. reported that among 233 patients encompassing 27 tumor types including CCA (*n* = 22), with deficient in DNA mismatch repair (dMMR) and with high microsatellite instability (MSI-H) treated with pembrolizumab 200 mg once every three weeks, the objective response rate was observed in 34.3% (95% CI, 28.3% to 40.8%) [105]. Median follow up was 13.4 months. Median progression-free survival was 4.1 months (95% CI, 2.4 to 4.9 months) and median overall survival was 23.5 months (95% CI, 13.5 months to not reached). Treatment-related adverse events occurred in 151 patients (64.8%). Thirty-four patients (14.6%) had grade 3 to 5 treatment-related adverse events, therefore exhibiting a safe profile for pembrolizumab. More generally, the safety profiles reported for ICI monotherapy in CCA are similar to those reported in other tumor types, without any apparent increase in rates of biliary complications or immune-related hepatitis in this at-risk population.

#### 4.1.2. Nivolumab

Nivolumab is a human immunoglobulin G4 (IgG4) monoclonal antibody that binds to the PD-1 receptor and blocks its interaction with PD-L1 and PD-L2. An ongoing phase 2 trial is aimed at exploring the role in advanced refractory biliary tract cancers [ClinicalTrials.gov Identifier: NCT02829918] [106]. Included patients failed or were intolerant to at least one line of therapy and no more than two lines of therapy. Participants received nivolumab at a dose of 240 mg intravenously every 2 weeks for 16 weeks and then 480 mg every 4 weeks from 17 weeks to the end of the study. The preliminary results [106] showed that 10 patients out of 45 (22%) achieved a partial response and 17 patients (37.8%) achieved stable disease. The disease control rate was 60%. All patients who responded were microsatellite stable. Nivolumab was well tolerated and has shown promising efficacy in refractory CCA including durable responses lasting two years. Another study aimed to assess the efficacy as well as the safety and tolerability of the immune checkpoint inhibitor nivolumab, as monotherapy or combined with chemotherapy [107] in 30 Japanese patients with biliary tract cancer. In the monotherapy cohort, median overall survival was 5.2 months (90% CI 4,5–8.7), median progression-free survival was 1.4 months (90% CI 1.4–1.4), and one of 30 patients had an objective response. In the combined therapy cohort, median overall survival was 15.4 months (90% CI 11.8-not estimable), median progression-free survival was 4.2 months (90% CI 2.8–5.6), and 11 of 30 patients had an objective response.

#### 4.1.3. Other Checkpoint Inhibitors

There are other ongoing trials exploring the role of other checkpoint inhibitors as durvalumab in solid tumors including CCA (i.e., ClinicalTrials.gov Identifier: NCT01938612).

In this phase 1 study preliminary results [108], the disease control rate at 12 weeks was 16.7% and 32.2%, in durvalumab (D) and in durvalumab plus tremelimumab (D + T), respectively. The median duration of response for the durvalumab cohort was 9.7 months and 8.5 months in the durvalumab with tremelimumab cohort. Median overall survival was 8.1 (95% CI, 5.6–10.1) months and 10.1 (95% CI, 6.2–11.4) months for durvalumab (D) and durvalumab plus tremelimumab (D + T) cohorts, respectively. Both therapies were tolerable for Asian patients, and no unexpected toxicities were observed.

#### 4.1.4. Combo-Strategies

Combinational strategies between different ICIs or a combination of an ICI with a chemo-therapy backbone seem to be a promising strategy that is also under exploration.

CCAs are known to be surrounded by a reactive tumor stroma, containing cancer-associated fibroblasts, endothelial cells, and immune cells, including tumor-associated macrophages (TAMs). These stromal elements produce soluble factors that play a role in modulating anticancer immune responses as reported by a small retrospective study involving 39 patients with CCA, in which high numbers of alternatively activated, ‘M2-like’ TAMs were reported to be associated with worse disease-free survival. Based on these observations, the combination of immunotherapy and microenvironmental targeting (i.e., granulocyte-macrophage colony-stimulating factor (GM-CSF) and pegylated IFNα-2b (Peg-IFNα-2b), fibroblast growth factor receptors (FGFR1–3) and heat-shock protein 90 (HSP90)) might be an effective treatment option and some ongoing trials are exploring this hypothesis.

Promising results have been reported through the administration of immunotherapies in malignancies commonly associated with viral infections [109] and the rationale for this might be the presentation of neoantigens associated with viral infections [110,111]. Liver-fluke disease, viral hepatitis B and C, and bacterial pyogenic cholangitis are all established risk factors for CCA [112], thus the subgroup of patients with CCA with underlying chronic viral infections or chronic inflammation, like sclerosing cholangitis, might take the most advantage by the administration of immunotherapy.

However, further studies are needed to draw more robust conclusions, particularly to define which subgroup of CCA might most benefit from immunotherapy and to identify specific predictors of tumor response.

The final results of the clinical trials studying ICIs in CCA setting are still ongoing. Even if the preliminary results have been quite modest when evaluating the nude overall response rate, these studies are still limited by the single-arm design, the small sample sizes, the unavailability of MSI/MMR status, and heterogeneous or unreported characteristics of the primary tumor, i.e., intrahepatic vs. extrahepatic or gallbladder.

On the other hand, the heterogeneity of CCA may represent a limit for evaluating the response rate, as the different tumors show differential responses to the same therapies. Therefore, targeting the immune system may be highly variable according to the different genetic profiles of the tumor and the microenvironment and sub-analyses of large cohorts of patients treated with ICIs are needed to identify the factors associated with response.

Despite a complex tumor and immune microenvironment with features suggesting the potential for antitumor immune responses, ICI monotherapy has shown limited efficacy in CCA to date, though the safety profile has been reassuring. Moreover, more complex approaches with combinations of different agents may lead to more clinical impact.

## 5. Future Perspective

Advanced CCA remains a difficult-to-treat disease. Future studies will continue focusing on therapies targeting specific genetic aberrations (FGFR2, IDH, BRAF, etc.) (Figure 2). We still need to understand which therapeutic molecules can give more therapeutic advances, especially in which subset of tumors presenting similar genomics and proteomics characteristics, thus suggesting specific and still unknown pathways that may influence response.

The value of molecular markers of the tumor in identifying these genetic aberrations also requires further study.

**Figure 3:**
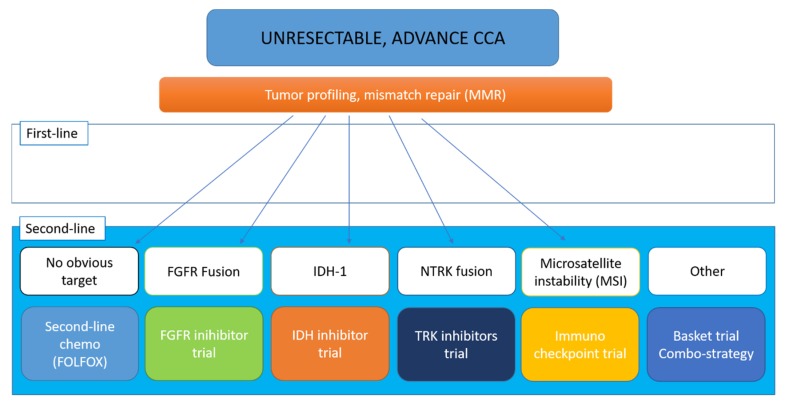
The landscape of targeted therapies for cholangiocarcinoma: current status and emerging targets.

The next generation of clinical trials—studying chemotherapy, target-therapy, immunotherapy or their combination aimed at getting better antitumor response are still ongoing (Figure 3).

## 6. Conclusions

Gemcitabine plus cisplatin remains the standard first-line systemic therapy for advanced CCA and offers a median survival of approximately one year. No standard regimens beyond the first line and no targeted or immunotherapy agents are approved yet in this disease. The development of molecular targeted therapy in this heterogeneous and relatively rare malignancy continues to be a challenging area. The rapidly growing precision medicine efforts have uncovered the underlying mutational landscape of this difficult to treat disease and paved the way for molecularly oriented clinical trials.

## Figures and Tables

**Table 1:** Summary of significant phase-III studies regarding chemotherapy for cholangiocarcinoma, in an adjuvant setting (A) and metastatic disease (M).

Study	Chemotherapy Regimen	Setting	Outcome	Reference
ESPAC-3	5-Fluoruracile versus gemcitabine	A	Better toxicity profile for gemcitabine	[10]
PRODIGE12-ACCORD18	GEMOX versus observation	A	RFS: No significant differences	[13]
BCAT	Gemcitabine versus observation	A	No significant differences	[14]
BILCAP	capecitabine versus observation	A	mOS: 51.1 months vs 36.4 months	[15]
ACTICCA-1	Capecitabine versus cisplatin/gemcitabine	A	ongoing	[16]
AC-02	Cisplatin/gemcitabine versus gemcitabine	M	mOS: 11.7 vs. 8.1 months	[18]
Phase III trial	GEMOX versus GEMOX plus erlotinib	M	mPFS: no differences	[21]
NuTide	Acelarin/cisplatin versus gemcitabine/cisplatin	M	ongoing	NCT04163900
ABC-06	FOLFOX versus ASC	M	Survival rate at 6 months: 50.6% vs 35.5%	[32]
TreeTopp	capecitabine versus capecitabin/Varlitinib	M	Ongoing	NCT03093870

GEMOX: Gemcitabine/oxaliplatin; FOLFOX: fluoruracile/oxaliplatin; ASC: active symptom control.

**Table 2:** Target agents in clinical development.

Target	Drug	Study Design	Significance	Ref. or Clinical Trial.gov
**IDH 1 and 2**				
IDH1 mutation	Ivosidenib	Phase III	Phase III trial evaluating the efficacy of AG120 in previously treated advanced CCA patients with IDH1 mutations (ClarIDHy)	Abou-Alfa ESMO 2019 [38]
**FGFR Selective and Non-selective Inhibitors**				
FGFR2	BGJ398	Phase II	Evaluate the activity of BGJ398 in patients with FGFR genetic alterations positive advanced CCA	NCT02150967
FGFR2	BGJ398	Phase III	Phase III trial evaluating the efficacy of BGJ298 + Cisplatin + Gemcitanine vs. Cisplatin + Gemcitabine alone in FGFR2 positive CCA	NCT03773302
FGFR pathway alterations	Erdafitinib	Phase I	Solid tumors/CCA	NCT02699606
FGFR mutation/Fusion	Derazantinib	Phase II	Evaluate the activity and the efficacy of derazantinib in patients with FGFR genetic alterations positive advanced iCCA	NCT03230318
FGFR mutation/Fusion	Derazantinib	expanded access	Investigational drugs outside of the clinical trial setting	NCT04087876
FGFR rearrangements/mutations	TAS-120	Phase I-II	Evaluate the efficacy in patients with FGFR gene rearrangements positive advanced iCAA	NCT02052778
FGFR rearrangements	TAS-120	Phase III/TAS-120 vs. Cisplatin Gemcitabine	Evaluate the efficacy in patients with FGFR gene rearrangements positive advanced iCCA	NCT04093362
FGFR1-2-3 Gene Alteration	Pemigatinib	Phase I	Evaluate the safety and MTD of Pemigatinib + Cisplatin + Gemcitabine in patients with advanced CCA	NCT04088188
FGFR2 rearrangement.	Pemigatinib	Phase III	Phase III trial evaluating the efficacy of Pemigatinib vs. Cisplatin + Gemcitanine in FGFR2 positive CCA	NCT03656536
**HER Family (ERBB2) Receptors**				
HER2	Trastuzumab	Phase II	Phase II trial evaluating the activity of trastuzumab in patients with HER2/neu-positive advanced gallbladder or CCA	NCT00478140
HER2	Trastuzumab Emanstine	Phase II	Phase II trial evaluating the activity of trastuzumab emanstine in patients with HER2/neu-positive advanced gallbladder or CCA	NCT02999672
**MAPK Pathway**				
BRAF	Dabrafenib and trametinib	Phase I	Evaluate the Activity and Safety of the combination regimen in subjects with BRAF V600E- Mutated Rare Cancers including CCA	NCT02699606
**Angiogenesis and Non-selective Kinase Inhibitors**				
VEGFR2	Ramucirumab	Phase II	Phase II trial evaluating the efficacy of Ramucirumab or Merestinib or Placebo Plus Cisplatin and Gemcitabine	NCT02711553
VEGFR2	Ramucirumab-Pembrolizumab	Phase I	Phase I trial evaluating the safety and the activity of Pembrolizumab and Ramucirumab in solid tumors	NCT02443324
**ROS1 and Neurotrophic Tyrosine Kinase Receptor (TRKA)**				
NTRK 1/2/3 (Trk A/B/C), ROS1, or ALK	Entrectinib	Phase II	Evaluate the Activity entrectinib in subjects with gene rearrangement of NTRK 1/2/3/ROS1/ALK in solid tumors including CCA	NCT02568267
Multiple Targets	Gemcitabine-Pazopanib	Phase II	Evaluate the Activity of Gemcitabine-Pazopanib in patients with advanced CCA	NCT01855724

^1^ CCA = cholangiocarcinoma. ^2^ iCCA = intrahepatic cholangiocarcinoma.
